# Modified Sick Neonatal Score and Delta: Modified Sick Neonatal Scores As Prognostic Indicators in Neonatal Intensive Care Units

**DOI:** 10.7759/cureus.28414

**Published:** 2022-08-25

**Authors:** Chaithra Padar, Aswathy Rajan, Ashvij Shriyan, Roshan A Oommen

**Affiliations:** 1 Department of Paediatrics, Indira Gandhi Institute of Child Health, Bengaluru, IND; 2 Department of Pediatrics, A.J. Institute of Medical Sciences, Mangaluru, IND

**Keywords:** msns, sick neonatal score, mortality risk scoring, neonatal emergencies, nicu

## Abstract

Background

Modified Sick Neonatal Score (MSNS) is a modification of the Sick Neonatal Score (SNS) by adding perinatal factors such as birth weight and gestational age to the scoring. A significantly higher sensitivity and specificity were obtained by adding the above parameters to SNS. The parameters in MSNS were simple and easy to score, but the scoring was done only once, and the utility of the score to assess the response to treatment was not analysed. In this study, we aimed to determine the role of MSNS as a prognostic indicator in the neonatal intensive care unit (NICU) and to study the correlation of the change in the MSNS (Delta-MSNS) 24 hours after admission with the outcomes and as a measure of response to treatment.

Methodology

A cross-sectional study was conducted for six months on all neonates admitted to the NICU during the study period. All babies were scored using MSNS containing eight basic clinical parameters, namely, respiratory rate, heart rate, axillary temperature, peripheral capillary refill time, random blood sugar, oxygen saturation, gestational age, and birth weight. Scoring by MSNS was done on the following two occasions: first score at admission, and second score 24 hours later. A change in the score during the first 24 hours of NICU stay (Delta-MSNS) was analysed for babies with an initial score of less than or equal to 12. All babies were followed up for analysis of outcomes, and outcomes were documented as discharge from the hospital or death. Length of hospital stay was documented for the babies that were discharged. Statistical analysis was done using the software RStudio v1.1.456. Kruskal-Wallis rank sum test was used to compare individual parameters of the score as well as the mean score between the expired and discharged groups. Spearman rank-order correlation coefficient was used to correlate the scores with length of hospital stay.

Results

A total of 248 neonates were considered for inclusion in the study. The mean score at admission was 7.94 (SD = 1.89) in the expired group and 14.46 (SD = 1.84) in the discharged group. The p-value for each of these was <0.001. Using MSNS as a test variable, a receiver operating characteristic (ROC) curve was generated, and as measured by the area under the curve was 0.98. A cut-off score of 10 was obtained that had a sensitivity of 88.24%, specificity of 95.2%, positive predictive value of 57.69%, and negative predictive value of 99%. A significant negative correlation was observed with a Spearman correlation coefficient of -0.67 when the initial MSNS was correlated with length of hospital stay in patients who were discharged. A significant negative correlation with a coefficient of -0.39 and p-value of 0.017 was determined when delta MSNS score was correlated with the discharged babies who had an initial score of less than 12.

Conclusions

MSNS is an easy-to-use bedside scoring system that requires minimal training and no invasive procedures. It has shown a high sensitivity in predicting mortality and length of hospital stay. Additionally, calculation of delta score was equally useful. It is a simplified score that enables providers to objectively assess the severity of illness with nominal training both in NICU as well as in low-resource settings.

## Introduction

The severity of neonatal illnesses has been assessed by several scoring systems. They are used to compare the performances of different newborn care units by risk adjustment, audit the severity of neonatal illness, determine the trends in results over time, and compare complications. However, they have also been used to give prognostic expectations to the parents [1].

A wide variety of scores ranging from simple to complex parameters have been used for decades. Clinical Risk Index for babies (CRIB), CRIB II, Berlin score, Neonatal Mortality Prognosis Index (NMPI), National Therapeutic Intervention Scoring System (NTISS), Score for Neonatal Acute Physiology (SNAP), SNAP-II, SNAPPE, SNAPPE-II, and many more have been investigated [1]. Even though scores with more clinical and laboratory parameters tend to be more accurate, they are difficult to complete and involve more expenses and skills.

A simpler score, Sick Neonate Score (SNS), was proposed in 2015 which used basic vital parameters [2]. A modification of SNS by adding perinatal factors such as birth weight and gestational age to the scoring was proposed in 2019 and was termed Modified Sick Neonatal Score (MSNS), which was studied in a sick newborn care unit (SNCU) [3]. Significantly higher sensitivity and specificity were obtained by adding the above parameters to SNS [3]. The parameters in MSNS were simple and easy to score, but the scoring was done only once, and the utility of the score to assess the response to treatment was not analysed.

This study was planned with the objective to determine the role of MSNS as a prognostic indicator in the neonatal intensive care unit (NICU) and to study the correlation of the change in the MSNS (Delta-MSNS) 24 hours after admission with the outcomes and as a measure of response to treatment.

## Materials and methods

This study was conducted at the level III NICU in a tertiary care centre following approval from the Institutional Ethical Committee of A.J. Institute of Medical Sciences (AJEC/REV/210/2019). It was a cross-sectional study conducted for six months from July 2019 to December 2019. All neonates admitted to the NICU during the study period were included, and the babies discharged against medical advice were excluded as the outcome could not be analysed. Informed consent was obtained from the parents of the neonates included in the study.

All babies were scored using MSNS (Table 1). MSNS is a simple score containing eight basic clinical parameters, namely, respiratory rate (RR), heart rate (HR), axillary temperature, peripheral capillary refill time (CRT), random blood sugar (RBS), oxygen saturation (SpO_2_), gestational age (GA), and birth weight. Each parameter has the best score of two and the least score of zero. The total maximum attainable score is 16, and the minimum score is zero. Axillary temperature was recorded using a digital thermometer, SpO_2_ by a pulse oximeter, RBS was done bedside using a glucometer, GA was calculated based on the last menstrual period, and birth weight was measured using an infant weighing scale.

**Table 1:** Parameters of Modified Sick Neonatal Score

Parameter/Score	0	1	2
Respiratory rate (cycles/minute)	Apnoea/grunt	>60	40–60
Heart rate (beats/minute)	<100	>160	100–160
Temperature (°C)	<36	36–36.4	36.5–37.5
Capillary refill time (seconds)	>5	3–5	<3
Random blood sugar (mg/dL)	<40	40–60	>60
Oxygen saturation (%)	<85	85–92	>92
Gestational age (weeks)	<32	32–36 (6/7)	≥37
Birth weight (kg)	<1.5	1.5–2.49	≥2.5

The Junior Residents posted at the NICU were trained to assess the above parameters. The details of the patient and the total MSNS score were documented in a semi-structured proforma. For babies who were brought intubated or on mechanical ventilation, the score for RR and SpO_2_ was taken as zero. MSNS score was calculated by adding the score for individual parameters. Scoring by MSNS was done on the following two occasions: the first score at admission, and the second score 24 hours later. A change in the score during the first 24 hours of NICU stay (Delta-MSNS) was analysed for babies with an initial score of less than or equal to 12 using the following formula: Delta-MSNS = MSNS score at admission - MSNS score after 24 hours.

All babies were followed up for analysis of outcomes, and outcomes were documented as discharge from the hospital or death. The length of hospital stay was documented for babies that were discharged.

Statistical analysis was done using the software RStudio v1.1.456. Kruskal-Wallis rank-sum test was used to compare individual parameters of the score, as well as the mean score between the expired and discharged groups. Spearman rank-order correlation coefficient was used to correlate the scores with the length of hospital stay. With MSNS as a variable, a receiver operating characteristic (ROC) curve was generated, area under the curve (AUC) was calculated, and a cut-off value for MSNS was obtained. The sensitivity, specificity, negative predictive value, and positive predictive values were calculated for the same.

## Results

A total of 262 neonates were admitted to the NICU during the study period. Among them, 12 were excluded as they were discharged against medical advice, and two babies were excluded due to congenital diaphragmatic hernia. In total, 248 neonates were finally considered for inclusion in the study. Informed consent was obtained from the parents of all participants enrolled in the study.

The baseline characteristics of the study population is summarised in Table 2. Overall, 35% of the cases were out born, 26% were preterm, and 32.7% had low birth weight. In total, 17 babies expired during the study period, which was consistent with the mortality rate in our setup.

**Table 2:** Baseline characteristics of the study population. LSCS: lower segment caesarean section

Baseline characteristics		Number (n)	%
Sex	Male	140	56
	Female	108	44
Place of birth	Inborn	160	65
	Out born	89	35
Mode of delivery	Vaginal	130	52
	LSCS	118	48
Gestational age (in weeks)	<32	14	5.65
	32–36	52	20.95
	≥37	182	73.3
Birth weight (in kg)	<1.5	16	6.5
	1.5–2.49	65	26.2
	≥2.5	167	73.3
Outcome	Death	17	6.9
	Discharge	231	93.1

The mean score at admission was 7.94 (SD = 1.89) in the expired group and 14.46 (SD = 1.84) in the discharged group. The p-value for each of these was <0.001.

**Figure 1:**
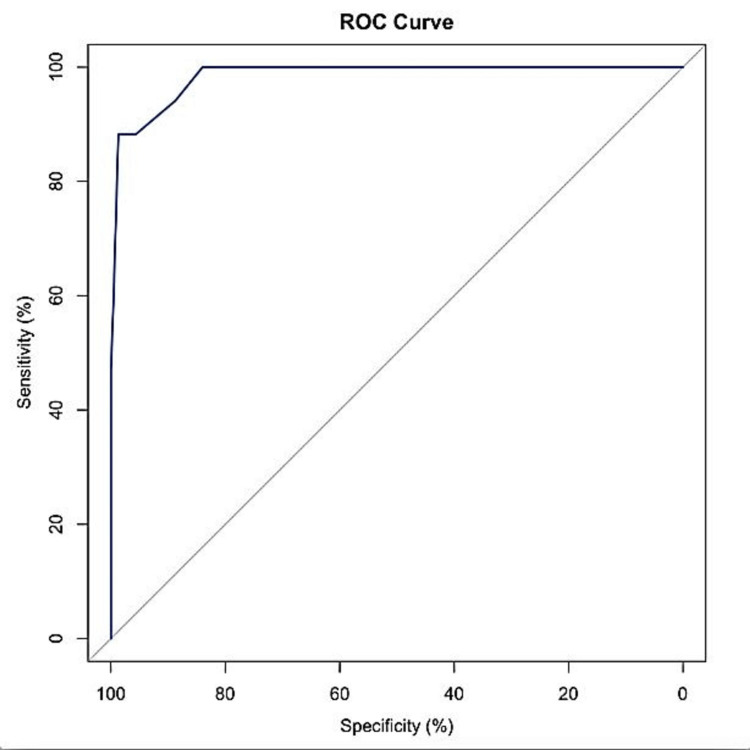
ROC curve showing an AUC of 0.98. ROC: receiver operating characteristic; AUC: area under the curve

Using MSNS as a test variable, an ROC curve was generated (see Figure 1). The overall discriminatory power of the total MSNS score, as measured by the AUC, was 0.98.

The cut-off score of 10 was obtained which had a sensitivity of 88.24%, specificity of 95.2%, positive predictive value of 57.69%, and negative predictive value of 99%.

**Figure 2:**
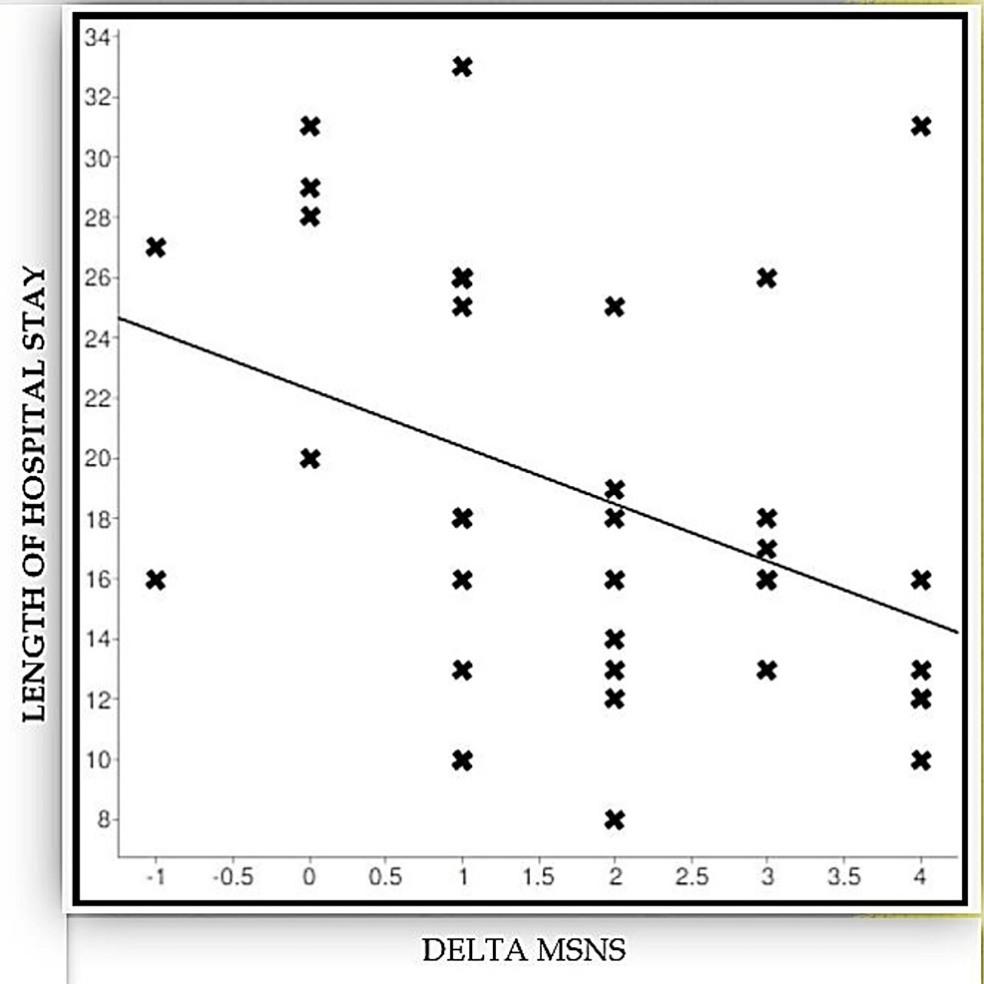
Correlation of Delta-MSNS with the length of hospital stay. Delta-MSNS: Delta-Modified Sick Neonatal Score

Initial MSNS was correlated with the length of hospital stay in patients who were discharged. A significant negative correlation was observed with a Spearman correlation coefficient of -0.67 (p < 0.001). Delta MSNS score was calculated and correlated (Figure 2) among the discharged babies who had an initial score of less than 12. Even this had a significant negative correlation with a coefficient of -0.39 and a p-value of 0.017.

## Discussion

Overall, 75% of neonatal deaths are known to occur in the first seven days of life [4]. Prompt recognition and intervention of disease severity are imperative in any attempts to reduce mortality and morbidity in critical care setups [5].

A variety of risk adjustment scores have been devised and validated over the years, each of which is used to compare outcomes across different NICUs [1]. A score that can efficiently predict subsequent clinical deterioration in sick newborns allows for early intervention and, in turn, reduction of mortality and morbidity in the NICU.

Among a total of 248 neonates included in this study, a mortality of 6.9% (n = 17) was encountered. The majority (73.3%) of the newborns were term babies with a birth weight of more than 2,500 g, with the rest of the parameters being of similar distribution. Mortality was higher in outborn babies (11.2%) and preterm babies (14.6%).

MSNS is a simple modification of SNS wherein there is no requirement for even a blood gas and is an advantage in a low-resource setting or when financial constraints have to be dealt with.

The mean MSNS at admission was 7.94 ± 1.89 among patients who died, whereas it was significantly higher in patients who were discharged at 14.46 ± 1.84 (p = 0.001). This is consistent with the study by Mansoor et al. who reported a mean MSNS of 8.22 ± 2.96 and 13.4 ± 2.14 in expired and discharged patients, respectively (p < 0.001) [3].

MSNS negatively correlated with the length of hospital stay, wherein a lower score indicated a longer hospital stay (r = - 0.67). Delta-MSNS was equally effective in predicting this. However, when the initial score is >12, one will be unable to analyse the change in score. Other scores such as SNAPPE II show a similar negative correlation with mortality (r = - 0.79) [6].

MSNS includes only basic objective parameters that can be accurately assessed with minimal training. Despite simplicity, our study demonstrated good specificity (95.24 %) and negative predictive value (99%), as well as fair sensitivity (88.24%).

Other commonly used scores include the SNS which showed only a sensitivity of 58.3% and a specificity of 52.7% for a score ≤8. The CRIB score that is used in preterms <32 weeks, when analysed for AUC, was found to be 0.87-0.90 [1].

More complex scores such as CRIB II, which is also done exclusively in preterms, include the use of base excess as a parameter for scoring. It shows a sensitivity of 94.9% and specificity of 82.4% for a score ≥11 [7]. Several other studies show the ROC curve plot of CRIB II, SNAPPE II, and Neonatal Mortality Risk Score (NMR-2000) to be 0.9, 0.849, and 0.88, respectively, all of which are comparable to our MSNS AUC [7-9].

Furthermore, a cut-off score of 10 correlated well with prognosis and prediction of mortality in our study. The highest specificity and sensitivity was derived to be a value of ≤10 which was a better predictor of mortality.

Limitations

A comparative analysis was not done in our study with other well-known established scores. In many resource-limited settings in India, the use of rectal temperature is not prevalent. The use of axillary temperature rather than rectal temperature in our study is an unreliable method to assess the temperature in newborns and may be considered to be a limitation. Another limitation is that the Delta score is not applicable in babies with an initial score of >12. The sample size was rather small to achieve a cut-off for the Delta score. Further research may be conducted to validate the MSNS, especially in low-resource settings and SNCUs, using a larger sample size. Moreover, its application in daily clinical decision-making needs to be investigated.

## Conclusions

MSNS is an easy-to-use bedside scoring system that requires minimal training and no invasive procedures. It has shown high sensitivity in predicting mortality and length of hospital stay. Additionally, the calculation of the Delta score was equally useful in the same. Further research may find the utility of delta-MSNS as a predictor of mortality compared to MSNS. Therefore, it is a simplified score that enables providers to objectively assess the severity of illness with nominal training both in NICU as well as low-resource settings.
